# Nonprescribed Antimicrobial Drugs in Latino Community, South Carolina

**DOI:** 10.3201/eid1106.040960

**Published:** 2005-06

**Authors:** Arch G. Mainous, Andrew Y. Cheng, Rebecca C. Garr, Barbara C. Tilley, Charles J. Everett, M. Diane McKee

**Affiliations:** * Medical University of South Carolina, Charleston, South Carolina, USA;; † Albert Einstein College of Medicine, Bronx, New York, USA

**Keywords:** antibiotics, respiratory infection, health beliefs and practices, Hispanic Americans

## Abstract

We investigated in a sample of Latinos the practices of antimicrobial drug importation and use of nonprescribed antimicrobial drugs. In interviews conducted with 219 adults, we assessed health beliefs and past and present behaviors consistent with acquiring antimicrobial drugs without a prescription in the United States. Many (30.6%) believed that antimicrobial drugs should be available in the United States without a prescription. Furthermore, 16.4% had transported nonprescribed antimicrobial drugs into the United States, and 19.2% had acquired antimicrobial agents in the United States without a prescription. A stepwise logistic regression analysis showed that the best predictors of having acquired nonprescribed antimicrobial drugs in the United States were beliefs and behavior consistent with limited regulations on such drugs. Many persons within the Latino community self-medicate with antimicrobial drugs obtained without a prescription both inside and outside the United States, which adds to the reservoir of antimicrobial drugs in the United States.

The amount of antimicrobial drugs consumed in a community is directly related to the amount of antimicrobial drug resistance found in the community (1,2). Inappropriate use of antimicrobial drugs, particularly for respiratory infections, has contributed to the major public health problem of antimicrobial resistance. Interventions in the United States have decreased inappropriate antimicrobial drug prescribing in general and for respiratory infections in particular (3–5).

In many nonindustrialized countries, antimicrobial resistance is an even greater problem than it is in the United States; however, many of these countries have few restrictions on the sale of antimicrobial agents, or laws are unevenly enforced (6–9). The dispensation of antimicrobial drugs without a prescription is particularly problematic in Latin America (9,10). Persons in countries with few regulations on antimicrobial agents tend to have cultural norms that encompass self-diagnosis and buying of antimicrobial drugs in subtherapeutic quantities (8,10,11). More than 320 million persons cross the border into the United States each year from Latin American countries where antimicrobial drugs are available without a prescription (12).

Health beliefs and practices are integrated into one's ethnic and cultural orientation (13–15). Limited evidence indicates that even after moving to the United States, persons from countries with limited or no restrictions on antimicrobial drugs are more likely than persons born in the United States to use them for inappropriate indications, such as common colds, and use them without a prescription (16,17).

Initiatives to increase judicious use of antimicrobial drugs to address antimicrobial resistance have focused primarily on changing prescribing patterns of US physicians and have failed to account for antimicrobial drug importation and concomitant inappropriate self-medication within immigrant communities. The pool of nonprescribed antimicrobial drugs entering the United States is currently unknown as is the extent of acquisition of nonprescribed antimicrobial drugs within communities whose health beliefs and practices may be inconsistent with those of the US public health community. Thus, the purpose of this study was to investigate a sample of Latinos living in the United States in terms of their practices of antimicrobial drug importation and acquisition and use of nonprescribed antimicrobial drugs.

## Methods

South Carolina, like many other cities and states that have historically had few Latinos, has experienced recent surges in immigration and now has small informal enclaves and social structures. Many Latinos in these new communities may lack documentation for US residency. Consequently, our sampling design took into account the fact that many probability-based sampling designs (e.g., random-digit dialing telephone survey, mail survey from a published list of addresses) would not adequately access this hard-to-reach population. Adult (≥18 years of age) Latinos attending 2 clinics in the greater Charleston, South Carolina, area that serve a primarily Latino population were approached for participation in a face-to-face, structured, confidential interview in which no names were recorded. This Latino population was employed primarily in agriculture.

Eligible persons included both those seeking care as well as those accompanying patients since the goal was to collect data from Latino adults regarding health behavior that was not necessarily linked to a current illness. Persons in the waiting rooms of the clinic were approached by an interviewer, who was introduced to the prospective respondent by clinic personnel. If persons did not consider themselves to be Latino, they were not included in the study. Data were collected in July and August 2004. The study was approved by the Medical University of South Carolina Institutional Review Board.

### Instrument

Previously used and validated surveys designed for use with Latino populations were accessed from the scientific literature and were consulted for question wording relevant to the present study's aims. An instrument was created in English, pretested initially in English for flow and comprehension, and following modifications was then translated into Spanish. Once in Spanish, the instrument was put through 2 levels of pretests. The first pretest was with medical center personnel who were fluent with Spanish and dealt with Latino populations. Following modifications from the first pretest, the second pretest was conducted with Latino persons seen at a clinic different from the ones used in the study to provide pretest experience with the instrument with a similar population. After the second pretest, the instrument was translated back into English so that the wording of the questions could be checked for consistency in both English and Spanish. Two trained bilingual interviewers administered the survey in a structured format in the participant's preferred language (Spanish or English) and reinforced to the potential respondents the anonymous nature of the survey.

### Variables

The study focused on 3 general sets of information. First, questions were included that assessed acquiring antimicrobial drugs without a prescription while outside the United States and corresponding self-medication. These questions were asked to get an idea of health behavior in their home culture and society. In addition, we asked whether the respondents believed that antimicrobial drugs should be available in the United States without a prescription. Second, questions were included that provided an assessment of the importation of nonprescription antimicrobial drugs into the United States and the context and circumstances related to this behavior. Third, a set of questions was included that assessed acquisition of nonprescribed antimicrobial drugs within the United States. We were specifically interested in the scope of acquisition of nonprescribed antimicrobial drugs from *bodegas*, *pharmacias*, or other stores since this behavior had been suggested in a study in New York City (17). For both importing nonprescribed antimicrobial drugs and acquiring them within the United States, a series of questions was asked, based on the pretest information, to gain an understanding of why the respondents would engage in these practices.

### Analysis

Initially, descriptive statistics were computed to gain an understanding of the extent of the acquisition and importation of nonprescribed antimicrobial drugs. We also computed chi-square values to examine bivariate relationships between importation of nonprescribed antimicrobial drugs and acquisition of nonprescribed antimicrobial drugs in the United States by health beliefs and behavior in the home country, access to care in the United States, and demographic characteristics. Finally, a stepwise logistic regression model was computed on the dependent variable of acquisition of nonprescribed antimicrobial drugs with the same set of variables (acquired antimicrobial drugs without a prescription outside the United States, believe antimicrobial drugs should be available in the United States without a prescription, health insurance, age, sex, education, time in United States, country of birth, health status) to determine the best predictors of this behavior. Because no one born in the United States had acquired nonprescribed antimicrobial drugs in the United States, the category variable of country of birth was not used as a predictor of acquiring nonprescribed antimicrobial drugs. Instead, country of birth was coded for inclusion in the regression as 1) born in Mexico or 2) born elsewhere.

## Results

A total of 277 adults were approached for participation. Four persons who were initially approached indicated that they did not consider themselves to be Latino. Of the remaining 273, 54 refused or provided incomplete information, leaving a sample of 219. The demographic characteristics of the sample are presented in Table 1.

**Table 1:** Characteristics of sample*

Characteristic	No. persons (%)
Country of birth	
Mexico	164 (74.8)
Other Central American country	24 (11.0)
South American country	24 (11.0)
United States (US)	7 (3.2)
Age, y (mean ± SD)	29.8 ± 8.1
Health status	
Excellent	30 (13.7)
Very good	64 (29.2)
Good	96 (43.8)
Fair	22 (10.1)
Poor	7 (3.2)
Sex	
Male	76 (34.7)
Female	143 (65.3)
Years in US	
<1	18 (8.2)
1–3	71 (32.4)
4–6	68 (31.1)
>6	62 (28.3)
Insurance	
None	200 (91.3)
Medicaid	14 (6.4)
Other	5 (2.3)
Education	
Did not graduate from high school	107 (48.9)
High school graduate or more	112 (51.1)

*N = 219.

A large proportion of the sample (30.6%) believed that antimicrobial drugs should be available in the United States without a prescription. The behavior of acquiring antimicrobial drugs without a prescription while outside of the United States was quite common, with 45.2% indicating that they had done this.

A substantial number of persons had transported nonprescribed antimicrobial drugs into the United States (16.4%). The primary illnesses for which they bought the antimicrobial drugs were primarily for what we believe to have been viral respiratory infections. Among respondents who reported bringing back antimicrobial drugs that they purchased without seeing a doctor first, the reported conditions they were trying to treat included cough (88.9%), ear infections (88.9%), sore throat (69.4%), and colds (58.3%). When asked whether, on their next trip outside the United States, they would purchase antimicrobial drugs without seeing a doctor first and bring them back into the country, 23.7% of the sample reported "likely" or "very likely." Among persons who transported nonprescribed antimicrobial drugs into the United States, the primary reason reported for doing so was because they had a mistrust of medicines in the United States and were more comfortable with medicines from the home country (30.6%); other reasons included the following: to pay less for medicines bought in the home country than they would for those bought in the United States (19.4%), to avoid going to the doctor while in the United States (16.7%), to avoid the language barrier to care in the United States (13.9%), to prepare for future illness (13.9%), and to treat someone else's medical problem (5.6%).

Acquiring antimicrobial drugs not prescribed for the respondent within the United States was also a common behavior (19.2%). Among those who acquired antimicrobial drugs in the United States without a prescription, 92.9% reported that they had acquired them without prescription at stores in the United States. As with transportation of nonprescribed antimicrobial drugs into the United States, the primary illnesses for which they acquired the drugs without a prescription were what we believe to have been viral respiratory infections. Among respondents who reported acquiring antimicrobial drugs without a prescription, they reported attempting to treat "gripe" (flu) (97.6%), ear infections (97.6%), cough (83.3%), and sore throat (80.9%). Additionally, 97.6% reported acquiring antimicrobial drugs to treat diarrhea. Among persons who had acquired antimicrobial drugs in the United States without a prescription, 64.3% suggested that doing so was preferable to going to the doctor, while 26.2% reported that it was cheaper than paying for the doctor visit in addition to paying for the prescription. Only 7.1% of this group reported acquiring antimicrobial drugs in this way because of language barriers.

Tables 2 and Table 3 show the relationship between home country behaviors, health beliefs, access to care variables, and demographic characteristics to importing nonprescribed antimicrobial drugs and acquiring nonprescribed antimicrobial drugs in the United States. Persons who acquired nonprescribed antimicrobial drugs in the United States had beliefs and practices consistent with limited regulations on antimicrobial drugs.

**Table 2:** Relationship between home country behavior, health beliefs, and access to care variables to importation of nonprescribed antimicrobial drugs into United States*

Variable	Brought antimicrobial drugs into United States, n (%)		
	Yes	No	p
Bought outside the United States without prescription			<0.01
Yes	27 (27.3)	72 (72.7)	
No	9 (7.5)	111 (92.5)	
Should antimicrobial drugs be available without prescription?			0.11
Yes	15 (22.4)	52 (77.6)	
No	21 (13.8)	131 (86.2)	
Age (y)			<0.01
<30	11 (9.2)	109 (90.8)	
≥30	25 (25.2)	74 (74.8)	
Health status			
Excellent-good	30 (15.8)	160 (84.2)	0.51
Fair-poor	6 (20.7)	23 (79.3)	
Sex			0.85
Male	13 (17.1)	63 (82.9)	
Female	23 (16.1)	120 (83.9)	
Years in United States			0.33
<4	12 (13.5)	77 (86.5)	
≥4	24 (18.5)	106 (81.5)	
Insurance			
None	33 (16.5)	167 (83.5)	0.94
Insured	3 (15.8)	16 (84.2)	
Education			
Did not graduate from high school	13 (12.2)	94 (87.8)	0.09
High school graduate or more	23 (20.5)	89 (79.5)	
Country of birth			
Mexico	24 (14.6)	140 (85.4)	0.13
Other Central American country	3 (12.5)	21 (87.5)	
South American country	8 (33.3)	16 (66.7)	
United States	1 (14.3)	6 (85.7)	

*N = 219.

**Table 3:** Relationship between home country behavior, health beliefs, and access to care variables to acquisition of nonprescribed antimicrobial drugs in United States*

Variable	Obtained in US without prescription, n (%)		
	Yes	No	p
Bought outside the United States without prescription			
Yes	31 (31.3)	68 (68.7)	<0.01
No	11 (9.2)	109 (90.8)	
Should antimicrobial drugs be available without prescription?			
Yes	25 (37.3)	42 (62.7)	<0.01
No	17 (11.2)	135 (88.8)	
Age (y)			
<30	29 (24.2)	91 (75.8)	0.04
≥30	13 (13.1)	86 (86.9)	
Health status			
Excellent-good	39 (20.5)	151 (79.5)	0.19
Fair-poor	3 (10.3)	26 (89.7)	
Sex			
Male	16 (21.0)	60 (79.0)	0.61
Female	26 (18.2)	117 (81.8)	
Years in United States			
<4	11 (12.4)	78 (87.6)	0.03
≥4	31 (23.8)	99 (76.2)	
Insurance			
None	40 (20.0)	160 (80.0)	0.32
Insured	2 (10.5)	17 (89.5)	
Education			
Did not graduate from high school	15 (14.0)	92 (86.0)	0.06
High school graduate or more	27 (24.1)	85 (75.9)	
County of birth			
Mexico	35 (21.3)	129 (78.7)	0.26
Other Central American country	2 (8.3)	22 (91.7)	
South American country	5 (20.5)	19 (79.2)	
United States	0	7 (100)	

*N = 219.

The best predictors of acquiring antimicrobial drugs in the United States without a prescription are shown in Table 4. Only 4 variables were significantly related. The strongest predictors were health beliefs and practices consistent with limited regulations on antimicrobial drugs.

**Table 4:** Significant predictors from stepwise logistic regression regarding whether person had obtained nonprescribed antimicrobial drugs in United States

Variable	Odds ratio	95% CI*
Should antimicrobial drugs be available without a prescription?		
Yes	5.70	2.52–12.92
No	1.00	–
Bought outside the United States without prescription		
Yes	5.41	2.33–12.58
No	1.00	–
Years in United States		–
<4	1.00	
≥4	4.28	1.72–10.68
Age (y)		
<30	3.00	1.28–7.01
≥30	1.00	–

*CI, confidence interval.

## Discussion

This study confirms the existence of a large reservoir of nonprescription antimicrobial drugs in the United States that are used for likely inappropriate self-medication in the Latino community. Besides being imported, many of these nonprescription drugs are acquired from small stores, showing an organized system of nonprescription antimicrobial drug distribution within the Latino community in the United States

The cultural beliefs and practice of obtaining antimicrobial drugs without prescriptions, particularly for what are likely viral respiratory infections, is reflected in the antimicrobial drug use patterns of the Latino community in this United States. Health beliefs and practices that were instilled in their countries of origin appear to be maintained even after living in the United States, as the relatively high frequency of acquisition of nonprescription antimicrobial drugs in the United States demonstrates. As previous research has shown, persons born in the United States are less likely to acquire antimicrobial drugs without a prescription within the United States (16). In fact, none of the Latino respondents born in the United States had acquired antimicrobial drugs without a prescription in the United States. Thus, a special emphasis with patient education should be made to target this population to instill health beliefs that are more consistent with those proposed by the US medical and public health communities.

An additional issue that may play a role in the ability to encourage appropriate use of antimicrobial drugs in this community are problems associated with access to health care. More than 90% of the respondents reported that they had no health insurance. Although health insurance was not a distinguishing variable, common reasons given by the respondents for both importation and acquisition of nonprescribed antimicrobial drugs in the United States revolved around the economics of doctor visits and costs of medication in the United States. A lack of health insurance may encourage Latinos to self-medicate with antimicrobial drugs.

Our findings should be interpreted within the context of several limitations to our study. First, the sample was recruited from a single mid-sized community, thereby limiting the generalizability of the results. However, many areas in the United States have seen recent large increases in the Latino population. These new communities may reflect different practices than communities that have had generations of Latino immigrants (e.g., Miami, New York, San Antonio). A second limitation concerns the location of data collection. By focusing on persons who sought treatment at a health clinic, Latinos who may not access care in the formal health care system would not be represented in this study. Those persons could be even more likely to acquire antimicrobial drugs without a prescription. A direction for future research would be to investigate these issues about antimicrobial drug use in a broader community sampling frame.

Third, the results are based on self-reports of behavior, some of which may be somewhat threatening to relate to an interviewer. Thus, the reports of antimicrobial drug importation and acquisition of nonprescribed antimicrobial drugs may be an underreport of actual behavior. Several strategies were used to try and obtain valid reports, including multiple pretests and having the clinic staff introduce the interviewers to the potential participants.

Persons may also have been confused about which medications are antimicrobial drugs. Following the pretests, the interview was modified to contain several instances of descriptions of antimicrobial drugs and names that would be recognizable to the Latino community. Moreover, each time a respondent reported acquiring antimicrobial drugs, the respondent was asked by the interviewer to name the drug to make sure that the respondent had actually acquired antimicrobial drugs. If in fact the results represent underreporting, the findings are even more dramatic because nearly 20% acknowledged getting antimicrobial drugs without a prescription in the United States.

Our findings suggest that a large public health problem is at hand. However, because this study is one of the first to document the problem and the first, to our knowledge, to specifically focus on both importation and US acquisition of nonprescribed antimicrobial drugs, government agencies have not yet responded to this problem. The South Carolina Department of Health and Environmental Control has a judicious antibiotic use initiative (South Carolina Careful Antibiotic Use, http://www.scdhec.gov/health/disease/sccause). Their initiatives are similar to many other initiatives that focus on both physician and patient education, but thus far have not focused on Latinos and nonprescription antimicrobial drug importation and local acquisition.

A substantial number of persons within the US Latino community self-medicate with antimicrobial drugs obtained without a prescription both inside and outside the United States, which adds to the reservoir of these drugs in the United States. The public health system and clinicians should be aware of the different health belief systems and practices in ethnic minority communities. Patient education materials should communicate in a culturally competent way the dangers of self-medication and antimicrobial misuse to both recent immigrants and others in the Latino community. US health policies also may need to be revised, with consideration given to tightening regulations to reduce the presence of nonprescribed antimicrobial drugs and to increasing access to care for many Latinos.
